# Melatonin affects hypoxia-inducible factor 1α and ameliorates delayed brain injury following subarachnoid hemorrhage via H19/miR-675/HIF1A/TLR4

**DOI:** 10.1080/21655979.2022.2027175

**Published:** 2022-02-16

**Authors:** Zhijian Xu, Fengduo Zhang, Hu Xu, Fan Yang, Gezhi Zhou, Minfeng Tong, Yaqiang Li, Song Yang

**Affiliations:** a Department of Neurosurgery, Affiliated Jinhua Hospital, Zhejiang University School of Medicine, Jinhua, Zhejiang, China; b Department of Emergency, Chinese People’s Army 971 Hospital, Qingdao, Shandong, China; c Department of Neurosurgery, Qingdao Fuwai Cardiovascular Hospital, Qingdao, Shandong, China; d Department of Neurosurgery, Shanghai East Hospital, School of Medicine, Tongji University, Shanghai, China; e Department of Neurosurgery, Jiaozhou Branch, Shanghai East Hospital, School of Medicine, Tongji University, Qingdao, Shandong, China

**Keywords:** Melatonin, TLR4, HIF1A, DBI following SAH, H19, miR-675, apoptosis

## Abstract

This study aimed to investigate the molecular mechanism of how melatonin (MT) interferes with hypoxia-inducible factor 1α (HIF1A) and toll-like receptor 4 (TLR4) expression, which is implicated in the management of delayed brain injury (DBI) after subarachnoid hemorrhage (SAH). Luciferase assay, real-time PCR, Western-blot analysis and immunohistochemistry (IHC) assays were utilized to explore the interaction among H19, miR-675, HIF1A and TLR4, and to evaluate the effect of MT on the expression of above transcripts in different groups. MT enhanced H19 expression by promoting the transcription efficiency of H19 promoter, and HIF1A was identified as a target of miR-675. HIF1A enhanced TLR4 expression via promoting the transcription efficiency of TLR4 promoter. Furthermore, administration of MT up-regulated miR-675 but suppressed the expressions of HIF1A and TLR4. Treatment with MT alleviated neurobehavioral deficits and apoptosis induced by SAH. According to the result of IHC, HIF1A and TLR4 protein levels in the SAH group were much higher than those in the SAH+MT group. Therefore, the administration of MT increased the levels of H19 and miR-675 which have been inhibited by SAH. In a similar way, treatment with MT decreased the levels of HIF1A and TLR4 which have been enhanced by SAH. MT could down-regulate the expression of HIF1A and TLR4 via the H19/miR-675/HIF1A/TLR4 signaling pathway, while TLR4 is crucial to the release of pro-inflammatory cytokines. Therefore, the treatment with MT could ameliorate post-SAH DBI.**Running title**: Melatonin ameliorates post-SAH DBI via H19/miR-675/HIF1A/TLR4 signaling pathways

## Introduction

Subarachnoid hemorrhage (SAH) is a disease in which the arterial blood flow points to the direction of subarachnoid space in the brain. The most frequent cause of SAH is ruptured aneurysm. SAH is a serious disorder with an annual incidence of 6–7 per 100,000 people and high mortality of 50% [1]. Treatment for SAH has primarily focused on the prevention of bleeding in the intracranial aneurysm, a factor contributing to early brain injury (EBI), as well as on the reversal of cerebral vasospasm, a critical cause of delayed brain injury (DBI) [2]. Specifically, it has been acknowledged that EBI, which occurs within the first 72 hours after ictus, is a potential risk factor of DBI, which led to poor prognosis of SAH [R301]. Moreover, EBI has been proved to one of the most relative factor which led to disability or even death in SAH patients [R302]. In fact, DBI and delayed cerebral ischemia following EBI are still considered as the most critical and preventable causes of poor prognosis in SAH, whereas inflammation has been considered as the key factor affecting the severity of brain injury [3].

Toll-like receptor 4 (TLR4) is highly expressed in the brain and can be activated by extravasated blood and damaged brain tissues upon the rupture of an intracranial aneurysm [4]. The inflammatory reaction and tissue damages caused by SAH can further activate TLR4, thus leading to EBI [3]. Previous studies have mainly concentrated on the role of the TLR4/nuclear factor kappa B (NF-κΒ) pathway in EBI, although no specific TLR4 antagonists have ever been tested for EBI [5]. After the stimulation of TLR4 with ligands, the myeloid differentiation factor 88 (MyD88)-dependent pathway can activate the nuclear factor (NF)-κΒ and synthesize pro-inflammatory cytokines or factors, including tumor necrosis factor (TNF)-α, interleukins such as interleukin-12 (IL-12), interleukin-8 (IL-8), interleukin-6 (IL-6) and interleukin-1beta (IL-1β), matrix metalloproteinase (MMP)-9, intercellular adhesion molecule (ICAM)-1, cyclooxygenases, superoxide, monocyte chemo-attractant protein (MCP)-1, nitric oxide and hydrogen peroxide [6].

As a type of small non-coding RNAs, microRNAs (miRNAs) play an essential role in gene regulation [7]. In fact, multiple roles of miRNA have been discovered in a wide range of physiological processes such as apoptosis, cell differentiation, cell growth, and carcinogenesis [8]. On the other hand, long non-coding RNAs (lncRNAs) are transcripts of more than 200 nt in length and are implicated in various critical cellular functions, such as RNA processing, chromatin modification, and gene regulation [9]. Previous articles have demonstrated that H19 expression is substantially increased upon cerebral I/R injury [10]. It has also been demonstrated that H19 can regulate post-SAH brain injury by mediating NGF- and P53-induced apoptosis through let-7a and microRNA −675 [11].

As a pharmacologic agent and a powerful antioxidant, melatonin (MT) can pass through the blood-brain barrier (BBB) easily and is associated with minimal toxicity even if it is consumed in large doses [12]. MT has been demonstrated to exert neuroprotective effects in animals suffering from neurologic injuries, including ischemic stroke and traumatic brain injury [13,14]. MT has been found to up-regulate the expression of H19, whereas miR-675 is located within the chromosomal segment of H19 [15,16]. Moreover, patients suffering from post-SAH EBI was found to typically experience abnormally enhanced inflammatory response, oxidative stress, cerebral vasospasm, and cell death [17], and a study by Yang et al., also demonstrated that MT treatment could substantially attenuate the neurological deficits while reducing the brain swelling in post-SAH EBI [18,19].

Furthermore, hypoxia-inducible factor 1α (HF1A) was found to be a target gene of miR-675 and could induce the expression of TLR4. Therefore, HIF1A may regulate the expression of miR-675 [20]. Our study aimed to investigate the molecular mechanism underlying the effect of MT upon HIF1A and TLR4 expression, which is implicated in the management of DBI after SAH. And based on the above evidences, we postulated that a possible signaling pathway of H19/miR-675/HIF1A/TLR4 might mediate the therapeutic effect of MT in the management of post-SAH DBI. To test such hypothesis, we established an animal model of SAH and treat it with MT. Subsequently, we investigated the involvement of the H19/miR-675/HIF1A/TLR4 signaling pathway in the effect of MT on the management of post-SAH DBI.

## Materials and methods

### Animals

A total of 36 adult male C57BL/6 J mice between ten to twelve weeks old were used in this study. These mice were purchased from the experimental animal center of our institute and weighed between 22 and 25 g. All experiments were carried out under NIH’s Guide for the Care and Use of Laboratory Animals and were approved by the Institutional Ethics Committee (Code: 160688M001).

### SAH model

An endovascular perforation method was used to establish the animal model of SAH as previously described [21,22]. In brief, 50 mg/kg pentobarbital sodium was injected to carry out anesthetization. A heat blanket was then used to maintain the rectal temperature of mice at 37 ± 0.5°C. The internal carotid artery, external carotid artery and left common carotid artery were then exposed through a midline incision at the neck, followed by ligation and dissection at the left external carotid to leave a 3-mm stump. A 5–0 nylon suture was then inserted into the left internal carotid artery to perforate the artery at the bifurcation of the anterior and middle cerebral arteries. The identical procedures were performed on sham-operated mice except for the artery perforation.

### Experimental protocol

The mice were assigned into the groups listed below: (1) SAH group (n = 12), (2) sham group (n = 12), (3) SAH + MT group (n = 12). For the MT group, MT was dissolved in 1% ethanol in 1 mL sterile saline, then intraperitoneally injected at a dose of 150 mg/kg and 12 h after SAH [23]. Schematics of the experiments are shown in Supplementary Figure 1.

### Neurological score

Neurological deficits were evaluated from 0 h to 96 h after the onset of SAH using an 18-point system containing six tests per previous published protocol [22,24]: forelimbs outstretching (0–3), spontaneous activity (0–3), climbing (1–3), symmetry in the movements of all limbs (0–3), response to vibrissae touch (1–3) and body proprioception (1–3). A higher score indicates an elevated function.

### RNA isolation and real-time PCR

Tissue and cell samples were treated with TRIZOL (Invitrogen, Carlsbad, CA) to extract total RNA, which was then converted to cDNA on a PE9600 PCR instrument (Perkin-Elmer, Foster City, CA). The mRNA expression of H19, miR-675, HIF1A, and TLR4 was then measured using real-time PCR using an SYBR Green kit following the manufacturer’s instructions. Accordingly, U6 and GAPDH were used as internal control. The thermocycle conditions were: 95°C for 5 min, 45 cycles of 95°C for 10s and 60°C for 50s, 90°C for 1 min, 55°C for 1 min, 95°C for 30s. All experiments were repeated in triplicated and the data was analyzed by the 2− ΔΔCt method [25]. The primers pairs used in this experiment were: H19-forward: 5’-TGCTGCACTTTA CAACCACTG-3’; H19-reverse: 5’-ATGGTGTCTTTGATGTTGGGC-3’; miR-675-forward: 5’-TGGTGCGGAGAGGGCCCACAGTG-3’; miR-675-reverse: 5’-GCGAGCACAGAATTAATACGAC-3’; HIF1A-forward: 5’-CGTGTTATCTGTCGCTTTGAGTC-3’; HIF1A-reverse: 5’-GTCTGGCTGCTGTAATAATGTTCC-3’; TLR4-forward: 5’-GGCATCATCTTCATTGTCCTTG-3’; TLR4-reverse: 5’-AGCATTGTCCTCCCACTCG-3’; U6-forward: 5’-CGCTTCGGCAGCACATATACTA-3’; U6-reverse: 5’-CGCTTCACGAATTTGCGTGTCA-3’; GAPDH-forward: 5’- CTTTGTCAAGCTCATTTCCTGG-3’, GAPDH-reverse: 5’-TCTTGCTCAGTGTCCTTGC-3’.

### Cell culture and transfection

U251 and SH-SY5Y cells were cultured at 37°C and in 95% humidity and 5% CO2 with a Dulbecco’s modified eagle medium (DMEM) (Invitrogen, Carlsbad, CA) containing 10% fetal calf serum (FCS) (Invitrogen, Carlsbad, CA). When the cultured cells grew to 90% adherence, they were passaged. Subsequently, the cell transfection was accomplished using Lipofectamine 2000 following the instruction of the manufacturer. For MT treatment, the cells were treated with 1 µm and 5 µm of MT for 48 h before the cells were collected for subsequent analyses [23].

### Luciferase assay

Full length 3’ UTR of HIF1A was amplified by PCR and cloned into a pGL3 vector (Promega, Madison, WI) downstream of the firefly luciferase reporter gene. Subsequently, a Quick-change site-directed mutagenesis kit (Stratagene, La Jolla, CA) was used to generate mutated 3’ UTR of HIF1A, which was also inserted to a pGL3 vector and used for subsequent transfection experiments. Similarly, miR-675 mimics were designed and synthesized before they were co-transfected to U251 and SH-SY5Y cells with either wild type (WT) or mutated 3’ UTR of HIF1A. At 48 h after transfection, the cells were harvested and the luciferase activity in transfected cells was measured using a dual-luciferase reporter assay (Promega, Madison, WI). In order to investigate the effect of HIF1A on the promoter of TLR4, the full length of TLR4 promoter was amplified by PCR and cloned into the pGL3 vector. Subsequently, HIF1A and TLR4 promoter were co-transfected to U251 and SH-SY5Y cells. At 48 h after transfection, the cells were harvested and the luciferase activity in transfected cells was measured using the dual-luciferase reporter assay.

### Western blot analysis

Tissue samples (basal cortical samples) were pooled and cell samples were lysed and resolved using 12% SDS-PAGE. Subsequently, the resolved proteins were blotted onto a membrane, which was then blocked at room temperature for 1 h with Tris-Buffered Saline and Tween 20 (TBST) containing 5% bovine serum albumin (BSA) and incubated at 4°C overnight with primary antibodies against HIF1A (ab92498, Abcam, Cambridge, MA) and TLR4 (ab8378, Abcam, Cambridge, MA). After TBST washing, the membrane was further incubated for 1 h at 37°C with HRP-labeled second antibodies (ab6728, Abcam, Cambridge, MA). Finally, after being developed using chemiluminescence reagents (Santa Cruz Biotech, Santa Cruz, CA), the optical density values of target proteins were measured.

### Apoptosis analysis

Treated U251 and SH-SY5Y cells were harvested by centrifugation. Subsequently, the cells were suspended in PBS to a concentration of 5–10 × 104 cells/mL and incubated with Annexin V-FITC and Propidium iodide. In the next step, the cell apoptosis profiles in different groups were analyzed using a FACSCanto II flow cytometer (BD, Franklin Lakes, NJ). The final apoptosis rate = early apoptosis rate + late apoptosis rate.

### Immunohistochemistry assay

Histochemical immunostaining assay was done using a streptavidin-peroxidase (SP) three-step method. All samples were first fixed in 4% formalin and embedded in paraffin. After antigen repair, the slides were incubated overnight at 4°C with primary antibodies against HIF1A (ab92498, Abcam, Cambridge, MA) and TLR4 (ab8378, Abcam, Cambridge, MA). After being washed repeatedly with PBS, the slides were incubated for 10 min at room temperature with the secondary antibody. Subsequently, the reaction was terminated by an anti-biotin-labeled peroxidase solution, and the slides were colorized with DAB. After re-staining with hematoxylin, the slides were dehydrated with anhydrous ethanol and dried before they were mounted in neutral gum and observed under a microscope.

### TUNEL assay

The frozen tissue samples were recovered at room temperature for 5–10s, followed by the addition of a resuscitation solution. After resuscitation, the samples were stained using a TUNEL kit (Beyotime, Shanghai, China). Subsequently, the samples were immersed in a 3% H2O2 solution at room temperature for 10 min and then rinsed with PBS for 5 min. After the addition of 50 uL of proteinase K solution (20 µg/mL), the samples were hydrolyzed for 20 min at room temperature to remove tissue proteins. On the next, the samples were washed with PBS and the antigen retrieval was done using a citrate buffer (0.01 M). Subsequently, the sections were cooled to room temperature and washed with PBS. Then, 50 μL of a TdT enzyme solution was added to the slices and incubated at 37°C for 1 h, whereas the reaction solution containing no TdT enzyme was used as a negative control. After the slices were washed with PBS, 50 μL of peroxidase-labeled anti-digoxigenin were added on the slides and incubated at 37°C for 30 min away from the light. Finally, a diamidino-2-phenylindole (DAPI) solution was added drop-wisely onto the slides at room temperature and incubated for 10 min before the slides were observed under a fluorescence microscope. The nuclei showing a green color were considered as apoptotic cells, while the nuclei showing a blue color were considered as normal cells. A total of 10 visual fields were selected randomly to calculate the apoptotic index.

### Statistical analysis

Statistical analysis was performed using SPSS16.0 statistical software. The measurement data were presented by mean values and standard deviation, while the average values between two groups were compared using t-tests. The comparison between multiple groups were conducted using one way ANOVA followed by the Tukey’s method as the post hoc test. And the P value of < 0.05 was considered statistically significant.

## Results

### MT enhanced H19 expression via promoting the transcription efficiency of H19 promoter

**Figure 1.**
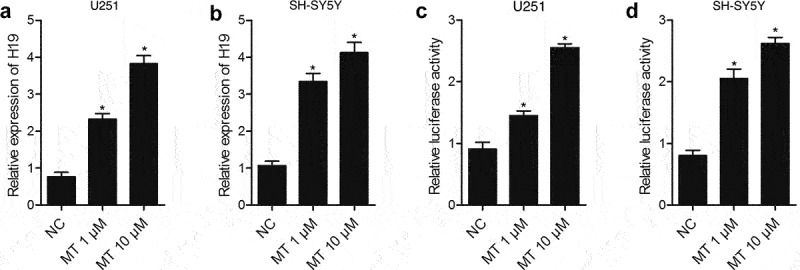
MT enhanced H19 expression via promoting the transcription efficiency of H19 promoter. (a) MT dose-dependently increased H19 level in U251 cells (* P value< 0.05, vs. NC; NC: negative control group). (b) MT dose-dependently increased H19 level in SH-SY5Y cells (* P value< 0.05, vs. NC; NC: negative control group). (c) MT dose-dependently increased the luciferase activity of H19 promoter in U251 cells (* P value< 0.05, vs. NC; NC: negative control group). (d) MT dose-dependently increased the luciferase activity of H19 promoter in SH-SY5Y cells (* P value< 0.05, vs. NC; NC: negative control group).

This study aimed to investigate the molecular mechanism of the therapeutic function of MET in the management of DBI after SAH. And we hypothesized that the expression of HIF1A and TLR4, which was mediated by H19, is associated with the therapeutic effect of MT in the treatment of post-SAH DBI. To test such hypothesis, we established an animal model of SAH and treat it with MT. Subsequently, we investigated the involvement of the H19/miR-675/HIF1A/TLR4 signaling pathway in the effect of MT on the management of post-SAH DBI. Real-time PCR and luciferase assay were performed to detect the effect of MT on H19 expression after various doses of MT (1 μM and 10 μM) were utilized to treat U251 and SH-SY5Y cells. As shown in Figure 1, the H19 level in U251 (Figure 1(a)) and SH-SY5Y (Figure 1(b)) cells treated with MT was up-regulated in a dose-dependent manner. Subsequently, a luciferase assay was performed to explore the underlying mechanism of MT effect on H19 expression. The results showed that the luciferase activity of H19 promoter in U251 (Figure 1(c)) and SH-SY5Y (Figure 1(d)) cells was increased in a dose-dependent manner after MT treatment, indicating that MT enhanced H19 expression via promoting the transcription efficiency of H19 promoter.

### HIF1A was a virtual target gene of miR-675

**Figure 2.**
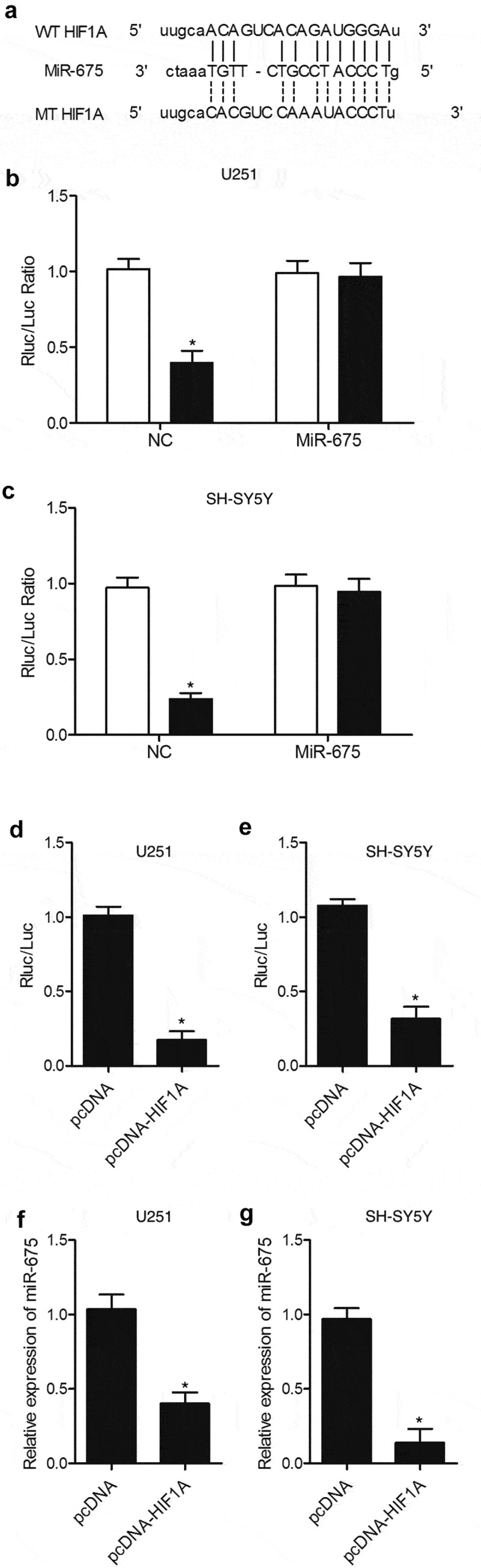
HIF1A was a virtual target gene of miR-675. (a) HIF1A was a potential target gene of miR-675 with a miR-675 binding site located in HIF1A 3ʹUTR. (b) MiR-675 mimic reduced the luciferase activity of U251 cells transfected with wild-type but not mutant HIF1A 3ʹUTR (* P value < 0.05, vs. wild-type HIF1A group). (c) MiR-675 mimic reduced the luciferase activity of SH-SY5Y cells transfected with wild-type but not mutant HIF1A 3ʹUTR (* P value < 0.05, vs. wild-type HIF1A group). (d) Transfection with HIF1A constructs reduced the luciferase activity of miR-675 promoter in U251 cells (* P value < 0.05, vs. pcDNA group). (e) Transfection with HIF1A constructs reduced the luciferase activity of miR-675 promoter in SH-SY5Y cells (* P value < 0.05, vs. pcDNA group). (f) MiR-675 level in U251 cells was down-regulated subsequent to transfection with HIF1A plasmids (* P value < 0.05, vs. pcDNA group). (g) MiR-675 level in SH-SY5Y cells was down-regulated subsequent to transfection with HIF1A plasmids (* P value < 0.05, vs. pcDNA group).

To identify the potential target gene of miR-675, we searched an online microRNA database (www.mirdb.org) and found that HIF1A was a potential target gene of miR-675 with a miR-675 binding site located in HIF1A 3ʹUTR (Figure 2(a)). Subsequently, a dual-luciferase assay was used to confirm that HIF1A was indeed a target gene of miR-675. As shown in Figure 2, the luciferase activity of U251 (Figure 2(b)) and SH-SY5Y (Figure 2(c)) cells transfected with wild-type HIF1A 3ʹUTR and miR-675 mimics was significantly lower than those cells transfected with the scramble control, while the transfection of miR-675 mimics showed no effect on the luciferase activity of mutant HIF1A 3ʹUTR, suggesting that HIF1A was a direct target gene of miR-675. The interaction between miR-675 and HIF1A was further investigated in U251 (Figure 2(d)) and SH-SY5Y (Figure 2(e)) cells transfected with HIF1A constructs, in which reduced luciferase activity of miR-675 promoter was observed compared with transfection of negative controls. In addition, the transfection with HIF1A constructs inhibited miR-675 expression in U251 (Figure 2(f)) and SH-SY5Y (Figure 2(g)) cells, validating that miR-675 and HIF1A mutually inhibited the expression of each other.

### HIF1A enhanced TLR4 expression via promoting the transcription efficiency of TLR4 promoter

**Figure 3.**
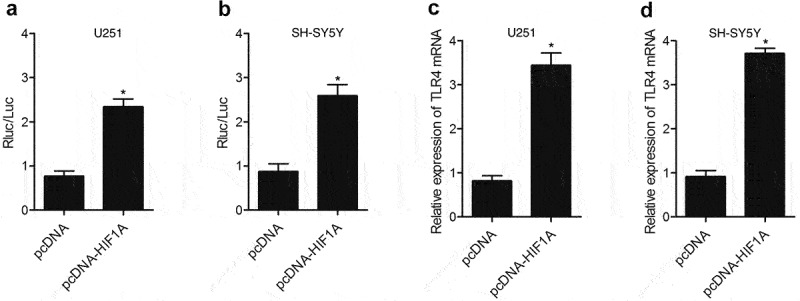
HIF1A enhanced TLR4 expression via promoting the transcription efficiency of TLR4 promoter. (a) Transfection with HIF1A constructs reduced the luciferase activity of TLR4 promoter in U251 cells (* P value < 0.05, vs. pcDNA group). (b) Transfection with HIF1A constructs reduced the luciferase activity of TLR4 promoter in SH-SY5Y cells (* P value < 0.05, vs. pcDNA group). (c) TLR4 level in U251 cells was down-regulated subsequent to transfection with HIF1A plasmids (* P value < 0.05, vs. pcDNA group). (d) TLR4 level in SH-SY5Y cells was down-regulated subsequent to transfection with HIF1A plasmids (* P value < 0.05, vs. pcDNA group).

Real-time PCR and luciferase assay were performed to detect the effect of HIF1A on TLR4 expression. As shown in Figure 3, the transfection of HIF1A constructs up-regulated the luciferase activity of TLR4 promoter in U251 (Figure 3(a)) and SH-SY5Y (Figure 3(b)) cells. In addition, the transfection of HIF1A constructs enhanced TLR4 expression in U251 (Figure 3(c)) and SH-SY5Y (Figure 3(d)) cells, validating the fact that HIF1A enhanced TLR4 expression by promoting the transcription efficiency of TLR4 promoter.

### Effect of MT on the expression of miR-675, HIF1A and TLR4

**Figure 4.**
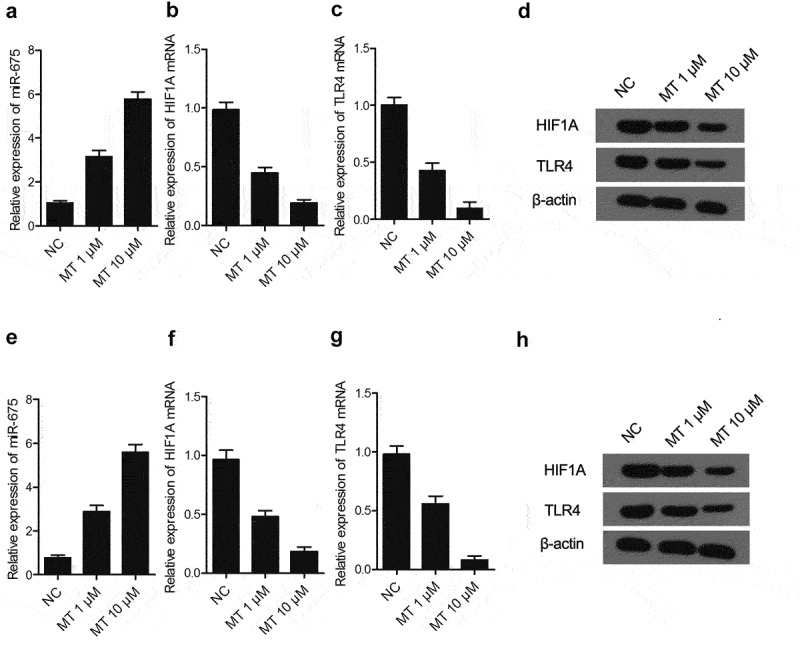
The effect of MT on the expression of miR-675, HIF1A and TLR4. (a) Treatment with MT elevated miR-675 level in U251 cells (* P value< 0.05, vs. NC; NC: negative control group). (b) The level of HIF1A mRNA in U251 cells was significantly decreased after MT treatment (* P value< 0.05, vs. NC; NC: negative control group). (c) The level of TLR4 mRNA in U251 cells was significantly decreased after MT treatment (* P value< 0.05, vs. NC; NC: negative control group). (d) HIF1A and TLR4 protein levels in U251 cells was down-regulated following MT treatment. (e) Treatment with MT elevated miR-675 level in SH-SY5Y cells (* P value< 0.05, vs. NC; NC: negative control group). (f) The level of HIF1A mRNA in SH-SY5Y cells was significantly dereased after MT treatment (* P value< 0.05, vs. NC; NC: negative control group). (g) The level of TLR4 mRNA in SH-SY5Y cells was significantly decreased after MT treatment (* P value< 0.05, vs. NC; NC: negative control group). (h) HIF1A and TLR4 protein levels in SH-SY5Y cells was down-regulated following MT treatment.

MiR-675, HIF1A and TLR4 expression were determined in U251 (Figure 4(a-d)) and SH-SY5Y (Figure 4(e-h)) cells after treatment with various doses of MT (1 μM and 10 μM). As shown in Figure 4, MT dose-dependently increased miR-675 (Figures 4(a-e)) expression, but reduced the mRNA levels of HIF1A (Figures 4(b-f)) and TLR4 (Figures 4(c-h)) in U251 (Figure 4(a-c)) and SH-SY5Y (Figure 4(e-g)) cells. In addition, MT treatment also reduced the protein levels of HIF1A and TLR4 (Figures 4(d-h)) in U251 (Figure 4(d)) and SH-SY5Y (Figure 4(h)) cells.

### MT attenuated SAH-induced neurobehavioral deficits and apoptosis

**Figure 5.**
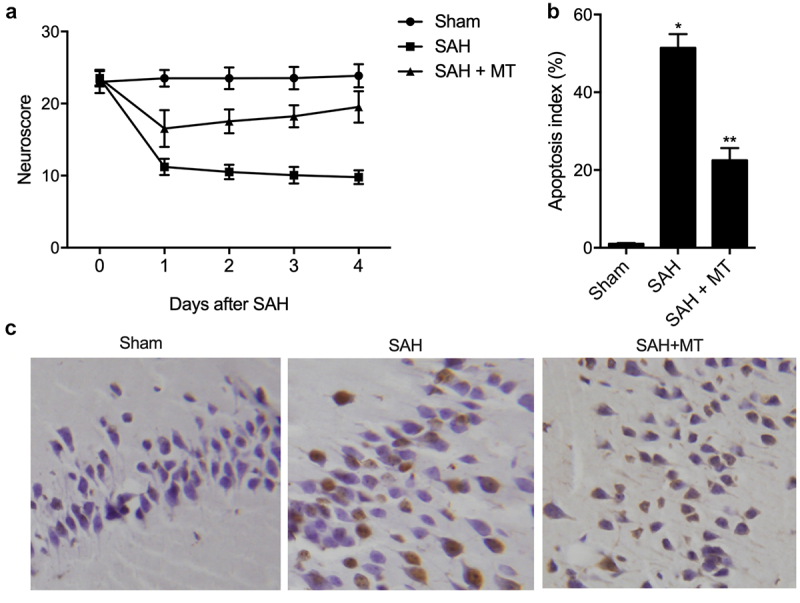
MT attenuated SAH-induced neurobehavioral deficits and apoptosis. (a) MT attenuated neurobehavioral deficits induced by SAH. (b/c) The extent of cell apoptosis in the SAH+MT group was much higher than that in the sham group, while the extent of cell apoptosis was the highest in the SAH group (Scale bar: 10 um; * P value< 0.05, vs. sham group; ** P value < 0.05, vs. SAH group).

Daily neurobehavioral testing was carried out at the baseline (before the onset of SAH) and after the onset of SAH using a neurological score test. As shown in Figure 5(a), vehicle-treated C57BL/6 J mice experienced significant neurobehavioral deficits after the onset of SAH, while MT-treated C57BL/6 J mice displayed significantly less neurobehavioral deficits. In addition, the extent of cell apoptosis (Figures 5(b-c)) was the lowest in the sham group and the highest in the SAH group. The severity of SAH in each SAH-involved group was comparable (data not shown).

### Differential expression of H19, miR-675, HIF1A and TLR4 among various groups

**Figure 6.**
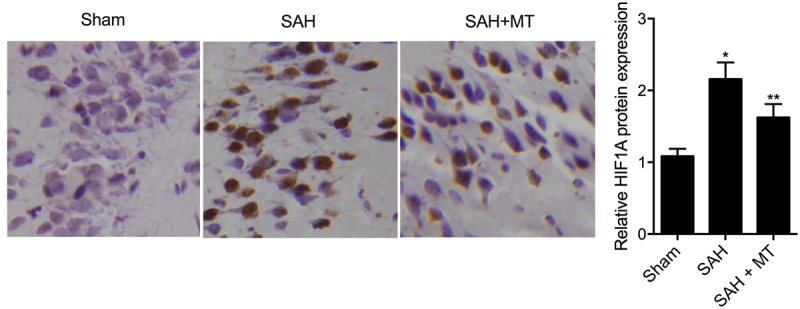
HIF1A protein level was the lowest in the sham group and the highest in the SAH group (Scale bar: 10 um).

**Figure 7.**
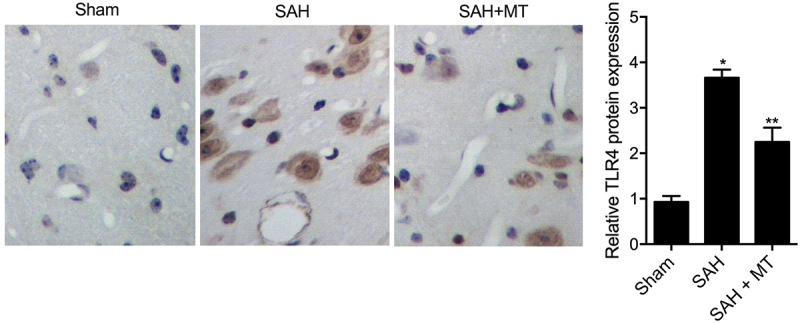
TLR4 protein level was the lowest in the sham group and the highest in the SAH group (Scale bar: 10 um).

**Figure 8.**
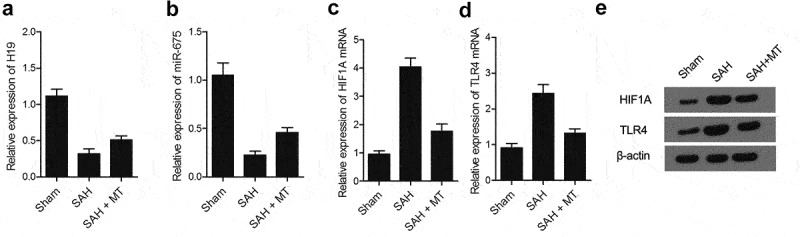
Differential expression of H19, miR-675, HIF1A and TLR4 among various groups. (a) H19 mRNA level was the highest in the sham group and the lowest in the SAH group (* P value< 0.05, vs. sham group; ** P value < 0.05, vs. SAH group). (b) miR-675 level was the highest in the sham group and the lowest in the SAH group (* P value< 0.05, vs. sham group; ** P value < 0.05, vs. SAH group). (c) HIF1A protein level was the lowest in the sham group and the highest in the SAH group (* P value< 0.05, vs. sham group; ** P value < 0.05, vs. SAH group). (d) TLR4 mRNA level was the lowest in the sham group and the highest in the SAH group (* P value< 0.05, vs. sham group; ** P value < 0.05, vs. SAH group). (e) Protein levels of HIF1A and TLR4 were the lowest in the sham group and the highest in the SAH group.

IHC assays were performed to measure the protein levels of HIF1A and TLR4 among sham, SAH, and SAH+MT groups. As shown in Figures 6 and 7, HIF1A (Figure 6) and TLR4 (Figure 7) protein levels were the highest in the SAH group and the lowest in the sham group. Subsequently, real-time PCR and Western-blot analysis were also utilized to measure the levels of H19, miR-675, HIF1A and TLR4 among sham, SAH, and SAH+MT groups. As shown in Figure 8, the mRNA levels of H19 (Figure 8(a)) and miR-675 (Figure 8(b)) were the lowest in the SAH group and the highest in the sham group. On the contrary, the levels of HIF1A (Figure 8(c-e)) and TLR4 (Figure 8(d,e)) were the highest in the SAH group and the lowest in the sham group. To sum up, MT could up-regulate the expression of H19, whose chromosomal segment hosted miR-675. And HF1A, which is also a target gene of miR-675, could induce the expression of TLR4, which played an important role in the release of pro-inflammatory cytokines such as TNF-α and interleukins. Inflammatory factors are involved in cell apoptosis, which is a significant manifestation of post-SAH DBI.

## Discussion

As a type of indolamine, MT is derived from tryptophan and has a high biological availability upon entering brain parenchyma [26]. Previous articles have demonstrated that MT exerts protective effects against post-SAH EBI in rats [27,28]. In addition, MT can be beneficial in alleviating EBI, and thus can exert a protective function in the treatment of SAH [29]. Furthermore, MT was also shown to reduce neuronal apoptosis, decrease mortality, and prevent vasospasm [30]. It was also demonstrated that MT could reduce focal cerebellum injury [31]. Moreover, the application of MT been also found that administration of MT could alter the expression of lncRNA and H19, and H19 has been shown to host miR-675, and miR-675 has been found to be a negative regulator of P53 [27,32]. A previous study also demonstrated that MT treatment could protect against post-SAH EBI by mediating the functions of H19-let-7a-NGF-apoptosis and H19-miR-675-P53-apoptosis signaling pathways [11,32]. And the carcinogenesis function of H19, which was identified as a miR-675 precursor, is also mediated by miR-675 [33]. In this study, we detected the effect of MT on H19 expression and found that MT enhanced H19 expression via promoting the transcription efficiency of H19 promoter. And we also demonstrated that MT could enhance the expression of miR-675.

It has been shown that the reduced expression of miR675-5p under low O2 partial pressure could inhibit hypoxic responses by reducing the expression of nuclear HIF-1α and miR675-5p [34]. In this study, we investigate the interaction between miR-675 and HIF1A, and found that miR-675 was directly bound to HIF1A, while the inhibitory effect between miR-675 and HIF1A were mutual. Subsequently, MT treatment also validated the interaction between miR-675 and HIF1A via inhibiting expression of HIF1A mRNA and protein while promoting expression of miR-675.

Interestingly, as the most frequently studied α subunit, stabilized HIF1A can migrate to the nuclei upon the reduction of oxygen tension. In the nuclei, HIF1A binds to ARNT/HIF1B and produce HIF1, which in turn interacts with hypoxic response elements to increase the expression of various genes and hence maintain the homeostasis in the body [35]. It is now recognized that both the accumulation of HIF1A and the transcriptional activation of HIF1 can be induced by inflammatory conditions and lipo-polysaccharide challenge [36]. In this study, we evaluated the effect of HIF1A on TLR4 expression, and found that HIF1A enhanced TLR4 expression via promoting the transcription efficiency of TLR4 promoter.

Moreover, as an important component of innate immunity, TLR4 plays an important role in the initiation of several signaling pathways, such as the release of NF-κB, and multiple proinflammatory mediators, including TNF-α, IL-8, IL-1β and IL-6 [37]. Kim et al. found that the presence of CoCl2 and severe hypoxia could increase TLR4 expression in macrophages [38]. In fact, as a well-studied pattern-recognition receptor (PRR), TLR can recruit specific adaptor molecules containing the TIR domain, including TRIF and MyD88, and initiate a series of signaling events such as the release of NF-κB and relevant cytokines [39]. In addition, TLR4 can interact with MyD88 and TIR-domain-containing adapter-inducing interferon-β (TRIF) to activate the NF-κB pathway, which in turn mediates the expression of inflammatory factors including IL-1α and IL-1β, IL-6 and TNF-α [40]. Previously, it has also been shown that TLR4 could lead to inflammatory injuries in the central nervous system during cerebral ischemia and infection [6]. In this study, we observed ameliorated neuro deficits and reduced apoptosis index along with inhibited TLR4 expression in the SAH animal models.

Inflammatory resolution is increasingly viewed as a process participated by multiple important mediators, and its dysregulation can lead to the onset of many chronic inflammatory diseases [41]. Apoptosis is shown to play a crucial function to maintain a normal state of inflammatory responses, whereas the dysregulation of inflammatory responses can be induced by an increased amount of reactive oxygen species (ROS), whose synthesis may also lead to excessive apoptosis [42–44]. While the cellular events involved in keeping a constant number of Leydig cells have not been clearly understood yet, apoptosis has been thought to play a critical function in the regulation of Leydig cells. In this study, MT treatment was shown to reduce miR-675 expression, while increasing the expression of HIF1A and TLR4. Furthermore, when treated with MT, the elevated HIF1A and TLR4 levels in SAH rats were partly restored.

## Conclusion

We firstly found that MT regulated HIF1A expression and ameliorated post-SAH DBI via the signaling pathway of H19/miR-675/HIF1A/TLR4. MT has also been found to up-regulate the expression of H19, whose chromosomal segment hosted miR-675. Furthermore, HF1A was found to be a target gene of miR-675 and induced the expression of TLR4, which played an important role in the release of pro-inflammatory cytokines such as TNF-α and interleukins. Moreover, inflammatory factors are involved in cell apoptosis, a significant manifestation of post-SAH DBI.

## Supplementary Material

Supplemental Material

## Data Availability

The data that support the findings of this study are available from the corresponding author upon reasonable request.

## References

[1] Listed N. Epidemiology of aneurysmal subarachnoid hemorrhage in Australia and New Zealand: incidence and case fatality from the Australasian cooperative research on subarachnoid hemorrhage study (ACROSS). Stroke. 2000;31:1843–1850. 10926945 10.1161/01.str.31.8.1843

[2] Suzuki H. What is early brain injury? Transl Stroke Res. 2015;6:1–3. 25502277 10.1007/s12975-014-0380-8

[3] Al-Mufti F, Amuluru K, Smith B, et al. Emerging markers of early brain injury and delayed cerebral ischemia in aneurysmal subarachnoid hemorrhage. World Neurosurg. 2017;107:148–159. 28755916 10.1016/j.wneu.2017.07.114

[4] Zheng B, Zhou X, Pang L, et al. Baicalin suppresses autophagy-dependent ferroptosis in early brain injury after subarachnoid hemorrhage. Bioengineered. 2021;12(1):7794–7804. 34704542 10.1080/21655979.2021.1975999 PMC8806453

[5] Kawakita F, Fujimoto M, Liu L, et al. Effects of Toll-Like receptor 4 antagonists against cerebral vasospasm after experimental subarachnoid hemorrhage in mice. Mol Neurobiol. 2017;54:6624–6633. 27738873 10.1007/s12035-016-0178-7

[6] Hanafy KA. The role of microglia and the TLR4 pathway in neuronal apoptosis and vasospasm after subarachnoid hemorrhage. J Neuroinflammation. 2013;10:83. 23849248 10.1186/1742-2094-10-83 PMC3750560

[7] Wang Z, Zuo G, Shi X-Y, et al. Progesterone administration modulates cortical TLR4/NF-kappaB signaling pathway after subarachnoid hemorrhage in male rats. Mediators Inflamm. 2011;2011:848309. 21403869 10.1155/2011/848309 PMC3051156

[8] Buchanan MM, Hutchinson M, Watkins LR, et al. Toll-like receptor 4 in CNS pathologies. J Neurochem. 2010;114:13–27. 20402965 10.1111/j.1471-4159.2010.06736.x PMC2909662

[9] Bartel DP. MicroRNAs: genomics, biogenesis, mechanism, and function. Cell. 2004;116:281–297. 14744438 10.1016/s0092-8674(04)00045-5

[10] Hwang HW, Mendell JT. MicroRNAs in cell proliferation, cell death, and tumorigenesis. Br J Cancer. 2006;94:776–780. 16495913 10.1038/sj.bjc.6603023 PMC2361377

[11] Gupta RA, Shah N, Wang KC, et al. Long non-coding RNA HOTAIR reprograms chromatin state to promote cancer metastasis. Nature. 2010;464:1071–1076. 20393566 10.1038/nature08975 PMC3049919

[12] Galano A, Tan DX, Reiter RJ. On the free radical scavenging activities of melatonin’s metabolites, AFMK and AMK. J Pineal Res. 2013;54:245–257. 22998574 10.1111/jpi.12010

[13] Yang S, Tang W, He Y, et al. Long non-coding RNA and microRNA-675/let-7a mediates the protective effect of melatonin against early brain injury after subarachnoid hemorrhage via targeting TP53 and neural growth factor. Cell Death Dis. 2018;9:99. 29367587 10.1038/s41419-017-0155-8 PMC5833397

[14] Barchas J, DaCosta F, Spector S. Acute pharmacology of melatonin. Nature. 1967;214:919–920. 6054984 10.1038/214919a0

[15] Beni SM, Kohen R, Reiter RJ, et al. Melatonin-induced neuroprotection after closed head injury is associated with increased brain antioxidants and attenuated late-phase activation of NF-kappaB and AP-1. FASEB J. 2004;18:149–151. 14597558 10.1096/fj.03-0323fje

[16] Pei Z, Fung PC, Cheung RT. Melatonin reduces nitric oxide level during ischemia but not blood-brain barrier breakdown during reperfusion in a rat middle cerebral artery occlusion stroke model. J Pineal Res. 2003;34:110–118. 12562502 10.1034/j.1600-079x.2003.00014.x

[17] Cai B, Ma W, Bi C, et al. Long noncoding RNA H19 mediates melatonin inhibition of premature senescence of c-kit+cardiac progenitor cells by promoting miR-675. J Pineal Res. 2016;61:82–95. 27062045 10.1111/jpi.12331

[18] Liu C, Chen Z, Fang J, et al. H19-derived miR-675 contributes to bladder cancer cell proliferation by regulating p53 activation. Tumour Biol. 2016;37:263–270. 26198047 10.1007/s13277-015-3779-2 PMC4841850

[19] Sehba FA, Hou J, Pluta RM, et al. The importance of early braininjury after subarachnoid hemorrhage. Prog Neurobiol. 2012;97:14–37. 22414893 10.1016/j.pneurobio.2012.02.003 PMC3327829

[20] Yang S, Tang W, He Y, et al. Long non-coding RNA and microRNA-675/let-7a mediates the protective effect of melatonin against early brain injury after subarachnoid hemorrhage via targeting tp53 and neural growth factor. Cell Death Dis. 2018;9(2):99. 29367587 10.1038/s41419-017-0155-8 PMC5833397

[21] Wang R, Zhou S, Wu P, et al. Identifying involvement of h19-mir-675-3p-igf1r and h19-mir-200a-pdcd4 in treating pulmonary hypertension with melatonin. Mol Ther Nucleic Acid. 2018;13:44–54. 10.1016/j.omtn.2018.08.015 PMC6146608 30240970

[22] Costa V, Raimondi L, Conigliaro A, et al. Hypoxia-inducible factor 1Alpha may regulate the commitment of mesenchymal stromal cells toward angio-osteogenesis by mirna-675-5P. Cytotherapy. 2017;19:1412–1425. 29111380 10.1016/j.jcyt.2017.09.007

[23] Schwartz AY, Masago A, Sehba FA, et al. Experimental models of subarachnoid hemorrhage in the rat: a refinement of the endovascular filament model. J Neurosci Methods. 2000;96(2):161–167. 10720681 10.1016/s0165-0270(00)00156-4

[24] Milner E, Johnson AW, Nelson JW, et al. HIF-1α mediates isoflurane-induced vascular protection in subarachnoid hemorrhage. Ann Clin Transl Neurol. 2015;2:325–337. 25909079 10.1002/acn3.170 PMC4402079

[25] Cao S, Shrestha S, Li J, et al. Melatonin-mediated mitophagy protects against early brain injury after subarachnoid hemorrhage through inhibition of nlrp3 inflammasome activation. Sci Rep. 2017;7(1):2417. 28546552 10.1038/s41598-017-02679-z PMC5445068

[26] Vellimana AK, Milner E, Azad TD, et al. Endothelial nitric oxide synthase mediates endogenous protection against subarachnoid hemorrhage-induced cerebral vasospasm. Stroke. 2011;42:776–782. 21317271 10.1161/STROKEAHA.110.607200 PMC3042520

[27] Livak KJ, Schmittgen TD. Analysis of relative gene expression data using real-time quantitative PCR and the 2(-Delta Delta C(T)). Meth Meth. 2001;25(4):402–408. 10.1006/meth.2001.1262 11846609

[28] Tordjman S, Chokron S, Delorme R, et al. Melatonin: pharmacology, functions and therapeutic benefits. Curr Neuropharmacol. 2017;15:434–443. 28503116 10.2174/1570159X14666161228122115 PMC5405617

[29] Wang Z, Ma C, Meng C-J, et al. Melatonin activates the Nrf2-ARE pathway when it protects against early brain injury in a subarachnoid hemorrhage model. J Pineal Res. 2012;53:129–137. 22304528 10.1111/j.1600-079X.2012.00978.x

[30] Yang GY, Betz AL, Chenevert TL, et al. Experimental intracerebral hemorrhage: relationship between brain edema, blood flow, and blood-brain barrier permeability in rats. J Neurosurg. 1994;81:93–102. 8207532 10.3171/jns.1994.81.1.0093

[31] Keniry A, Oxley D, Monnier P, et al. The H19 lincRNA is a developmental reservoir of miR-675 that suppresses growth and Igf1r. Nat Cell Biol. 2012;14:659–665. 22684254 10.1038/ncb2521 PMC3389517

[32] Gao Y, Wu F, Zhou J, et al. The H19/let-7 double-negative feedback loop contributes to glucose metabolism in muscle cells. Nucleic Acids Res. 2014;42:13799–13811. 25399420 10.1093/nar/gku1160 PMC4267628

[33] Ersahin M, Toklu HZ, Çetinel Ş, et al. Melatonin reduces experimental subarachnoid hemorrhage-induced oxidative brain damage and neurological symptoms. J Pineal Res. 2009;46:324–332. 19215574 10.1111/j.1600-079X.2009.00664.x

[34] Martinez-Cruz F, Espinar A, Pozo D, et al. Melatonin prevents focal rat cerebellum injury as assessed by induction of heat shock protein (HO-1) following subarachnoid injections of lysed blood. Neurosci Lett. 2002;331:208–210. 12383932 10.1016/s0304-3940(02)00884-4

[35] Matouk IJ, Halle D, Gilon M, et al. The non-coding RNAs of the H19-IGF2 imprinted loci: a focus on biological roles and therapeutic potential in Lung Cancer. J Transl Med. 2015;13:113. 25884481 10.1186/s12967-015-0467-3 PMC4397711

[36] Lo Dico A, Costa V, Martelli C, et al. MiR675-5p acts on HIF-1alpha to sustain hypoxic responses: a new therapeutic strategy for glioma. Theranostics. 2016;6:1105–1118. 27279905 10.7150/thno.14700 PMC4893639

[37] Semenza GL. Oxygen sensing, homeostasis, and disease. N Engl J Med. 2011;365:537–547. 21830968 10.1056/NEJMra1011165

[38] Richard DE, Berra E, Pouyssegur J. Nonhypoxic pathway mediates the induction of hypoxia-inducible factor 1alpha in vascular smooth muscle cells. J Biol Chem. 2000;275:26765–26771. 10837481 10.1074/jbc.M003325200

[39] Akira S, Uematsu S, Takeuchi O. Pathogen recognition and innate immunity. Cell. 2006;124:783–801. 16497588 10.1016/j.cell.2006.02.015

[40] Kim SY, Choi YJ, Joung SM, et al. Hypoxic stress up-regulates the expression of Toll-like receptor 4 in macrophages via hypoxia-inducible factor. Immunology. 2010;129:516–524. 20002786 10.1111/j.1365-2567.2009.03203.x PMC2842498

[41] Kawai T, Akira S. The role of pattern-recognition receptors in innate immunity: update on Toll-like receptors. Nat Immunol. 2010;11:373–384. 20404851 10.1038/ni.1863

[42] Miyake K. Endotoxin recognition molecules MD-2 and toll-like receptor 4 as potential targets for therapeutic intervention of endotoxin shock. Curr Drug Targets Inflamm Allergy. 2004;3:291–297. 15379597 10.2174/1568010043343633

[43] Shores DR, Binion DG, Freeman BA, et al. New insights into the role of fatty acids in the pathogenesis and resolution of inflammatory bowel disease. Inflamm Bowel Dis. 2011;17:2192–2204. 21910181 10.1002/ibd.21560 PMC4100336

[44] Oyinloye BE, Adenowo AF, Kappo AP. Reactive oxygen species, apoptosis, antimicrobial peptides and human inflammatory diseases. Pharmaceuticals (Basel). 2015;8:151–175. 25850012 10.3390/ph8020151 PMC4491653

