# Supplementary figures and images for: Melatonin affects hypoxia-inducible factor 1α and ameliorates delayed brain injury following subarachnoid hemorrhage via H19/miR-675/HIF1A/TLR4
Source: Bioengineered. 2022 Feb 16;13(2):4235–47. doi: 10.1080/21655979.2022.2027175 (PMC8974079; doi:10.1080/21655979.2022.2027175)

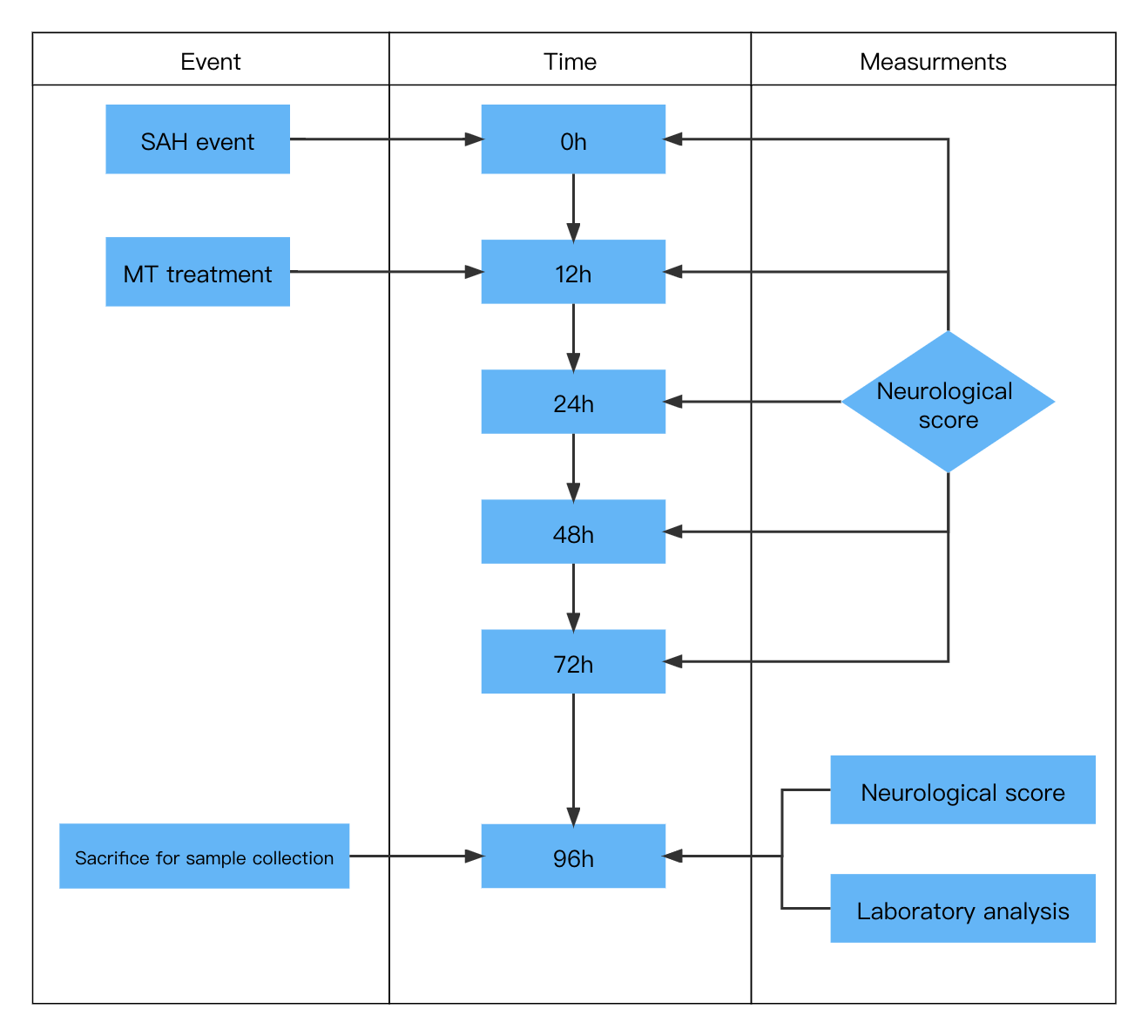

Supplement: Supplemental Material [file KBIE_A_2027175_SM8185.zip › supplementary/Schematics of the experiments.jpeg]
